# Young South African Women on Antiretroviral Therapy Perceptions of a Psychological Counselling Program to Reduce Heavy Drinking and Depression

**DOI:** 10.3390/ijerph17072249

**Published:** 2020-03-27

**Authors:** Petal Petersen Williams, Carrie Brooke-Sumner, John Joska, James Kruger, Lieve Vanleeuw, Siphokazi Dada, Katherine Sorsdahl, Bronwyn Myers

**Affiliations:** 1 Alcohol, Tobacco and Other Drug Research Unit, South African Medical Research Council, Francie van Zyl Drive, Tygerberg 7505, South Africa; Carrie.Brooke-sumner@mrc.ac.za (C.B.-S.); Siphokazi.Dada@mrc.ac.za (S.D.); Bronwyn.Myers@mrc.ac.za (B.M.); 2 Department of Psychiatry and Mental Health, University of Cape Town, Cape Town 7700, South Africa; 3 Alan J Flisher Centre for Public Mental Health, Department of Psychiatry and Mental Health, University of Cape Town, Cape Town 7700, South Africa; Katherine.Sorsdahl@uct.ac.za; 4 HIV Mental Health Research Unit, Division of Neuropsychiatry, Department of Psychiatry and Mental Health, University of Cape Town, Cape Town 7700, South Africa; john.joska@uct.ac.za; 5 Western Cape Department of Health., 8 Riebeeck Street, Cape Town 8000, South Africa; James.Kruger@westerncape.gov.za; 6 Health Systems Research Unit, South African Medical Research Council, Francie van Zyl Drive, Tygerberg 7505, South Africa; Lieve.vanLeeuw@mrc.ac.za

**Keywords:** young women, hazardous alcohol use, depression, HIV, adherence, MI-PST counselling, psychosocial intervention

## Abstract

Young women in South Africa remain most at risk for HIV infection. Several factors contribute to the high incidence rate in this population, including hazardous drinking and depression. Addressing common mental disorders (CMDs) such as depression and alcohol use disorders is key to effective HIV treatment. We explored the experiences and perceptions of young South African women on antiretroviral therapy (ART) of a lay health worker (LHW)-delivered psychosocial intervention based on motivational interviewing (MI) and problem-solving therapy (PST) to reduce heavy drinking and depression. We conducted 27 in-depth interviews with young women (aged 18–35) recruited from 16 primary care clinics in the Western Cape province of South Africa. Discussion topics included young women’s life experiences leading to their enrollment in the program, their perceptions of the counselling sessions and the quality of their interaction with the counsellor. Qualitative data were analyzed using a framework approach. The findings highlighted the impact adverse life experiences and stressful life circumstances have on young women’s use of alcohol and symptoms of depression and the effect this has on ART adherence. The findings suggest that women found the intervention components that helped them develop strategies for coping with their past experiences, managing current life stressors, and regulating negative thoughts and emotions most beneficial. Taken together, these findings confirm the acceptability of LHW-delivered MI-PST counselling for this population, but suggest that the relevance of the MI-PST intervention for this highly vulnerable population could be further enhanced by including a focus on psychological trauma.

## 1. Introduction

Sub-Saharan Africa remains disproportionately affected by HIV, accounting for more than 70% of the global burden of disease [1]. In this region, rates of new infection are concentrated among young women [2], with incidence rates up to eight-fold higher among adolescent girls and young women aged 15–24 years compared with their male peers [3]. HIV incidence remains high despite efforts to ensure that the virus remains undetectable and therefore untransmittable among people living with HIV (PLWH) by scaling up antiretroviral therapy (ART). 

Several factors contribute to these high incidence rates among adolescent girls and young women (AGYW). Gender-based violence including sexual assault in South Africa is rife and is linked to HIV incidence and psychological trauma [4]. These experiences of trauma may be linked to heavy drinking and other common mental disorders, such as depression [5]—factors known to impact on women’s adherence to ART. As sub-optimal adherence to ART contributes to continued infectivity and onward transmission among PLWH, with young women less likely to be retained on ART than older women [6,7], there have been calls to improve AGYW’s engagement in HIV care and adherence to ART as part of a comprehensive strategy towards eliminating HIV in South Africa [8,9]. 

Although heavy drinking and common mental disorders (CMDs) are highly prevalent barriers to ART adherence among PLWH in South Africa [10,11,12,13,14,15,16,17], they are rarely addressed as part of HIV treatment [18]. Routine screening for heavy drinking and CMDs does not occur and often goes undetected in these services. This is a concern given evidence that integrating substance use and mental health services into HIV care can improve care engagement [19,20]. This is partly due to a lack of mental health services at the primary care level. Mental health services that are available are geared towards medication management for severe mental illnesses, with psychological counselling for CMDs rarely being provided [12,21]. To address the human resource and financial constraints that limit the provision of mental health services within primary care in South Africa and other low-and middle-income countries [21,22], task-sharing approaches (in which lay health workers (LHW) are trained to deliver psychosocial interventions with the support of more specialist staff) are being promoted as feasible, acceptable and cost-effective approaches to deliver basic mental health counselling and support services within the context of limited human resources for mental health [23]. While South Africa’s health policies support the provision of task-shared psychological counselling within the context of HIV care, there is uncertainty about how to configure resources within the primary healthcare system to allow for LHW-delivered psychological interventions. To guide health service planners in their decisions regarding how to proceed with integrating LHW-led psychological counseling, we embarked on a trial of two different approaches to integration, known as Project MIND [24]. In both approaches, LHWs delivered a structured intervention consisting of three structured counselling sessions based on motivational interviewing (MI) and problem-solving therapy (PST), with the option of a fourth booster session. 

While this intervention has been tested in other patient populations who use substances in South Africa [25,26,27], little is known about the extent to which young South African women on ART perceive the MI-PST interventions as acceptable and beneficial for addressing both alcohol use and depression. Understanding young women’s perceptions and experiences of these interventions could identify modifications to the intervention content or delivery mechanisms that are needed to better meet young women’s psycho-social needs and intervention preferences [28]. Findings from this study can inform future interventions with young women, such as the South African Medical Research Council’s Social Impact Bond, which aims to improve outcomes related to HIV among school-going adolescent girls and young women in South Africa. 

This paper begins to address this gap by exploring young South African women on ART’s experiences and perceptions of an LHW-delivered counselling program to reduce heavy drinking and risk of depression with a view to understanding and identifying ways to increase the acceptability and uptake of the intervention. 

## 2. Methods

This qualitative study investigated the counselling experiences of young women living with HIV who were enrolled into the Project MIND trial. Trial procedures, setting and context are fully described elsewhere [24]. We present this study in line with COnsolidated criteria for REporting Qualitative research (COREQ) guidance for reporting qualitative research [29]. 

Between May 2018 and March 2019, we recruited 801 PLWH from 24 primary care clinics in the Western Cape province of SA. These facilities were stratified by urban–rural status before being randomly assigned to either treatment as usual or one of two intervention arms. During the recruitment period, health providers asked all patients presenting for routine HIV treatment about their recent alcohol use and mood. Patients who reported alcohol use and low mood were referred to a study assessor who described the study before requesting verbal consent for eligibility screening. Eligibility criteria included (i) being at least 18 years old; (ii) taking antiretroviral therapy (ART) for HIV; (iii) screening positive for hazardous drinking on the Alcohol Use Disorders Identification Test (AUDIT) (AUDIT ≥ 8) [30] or depression with the Center for Epidemiologic Studies Depression Scale (CES-D ≥ 16) [31]; and (iv) providing consent to all study procedures. Patients were excluded from study participation if they were receiving medication or counselling for a mental health problem such as depression, anxiety or other mental illness. If eligible patients were interested in study participation, an appointment was made for an enrolment visit. At this visit, the assessor confirmed the participant’s eligibility prior to obtaining their consent for trial participation. The assessor then conducted a computer-assisted personal interview with consenting participants in their choice of English, Afrikaans or isiXhosa (the three official languages of the province). These interviews included questions on the participant’s socio-demographic characteristics, HIV treatment and engagement in care, CMDs, and psychosocial factors that contribute to CMDs. Participants also provided blood samples for HIV viral load testing to assess the extent to which their HIV was controlled. The assessor then scheduled counselling appointments for participants recruited from facilities assigned to the intervention conditions. 

Participants recruited from the 16 facilities assigned to one of the two intervention arms were offered a lay counsellor-delivered MI-PST intervention, with MI elements focused on building readiness for behavioral change and engagement in counselling, whereas the PST content focused on helping patients cope with life stressors and negative emotions and to accept problems that cannot be solved [24,32]. Table 1 provides a summary of the content of the intervention sessions. Participants were given six weeks to complete the three-session intervention, and an additional two weeks to complete the fourth optional session. Participants were tracked for follow-up assessments at 6 and 12-months post-enrolment, at which time the baseline questionnaire was re-administered, and blood samples were drawn for repeat viral load testing. 

For this study, we were interested in exploring the counselling experiences of young women (18–35 years of age) living with HIV. We drew up a list of all participants who met these criteria (n = 260) and randomly selected a sub-set to invite for a 13-month post-enrolment interview. In total, 53 women from seven sites were contacted of which 27 agreed to an interview. Those who declined the offer of an interview cited work and not having time as the main reasons. Baseline AUDIT (alcohol) and CES-D (depression) scale scores as well as treatment completion rates did not differ between those who agreed and those who declined to be interviewed. Amongst those who agreed to participate, three completed one counselling session, five completed two sessions, 19 completed all three sessions and three received the booster session. 

In-depth interviews were conducted by research assistants in a private space at the facility from which young women had been recruited. These research assistants had postgraduate qualifications, training in qualitative research methods, experience in conducting interviews, and were proficient in the local languages. They were not known to the participants prior to the interviews. The study was explained in the participants’ first language, and written informed consent was obtained before conducting the interview. Interviews were guided by an interview schedule with open-ended questions that aimed to elicit information about young women’s experiences leading to their enrollment in the program, as well as regarding the counselling sessions and quality of interaction with the counsellor (See Appendix A, interview schedule). Interviews were digitally audio-recorded before being transcribed verbatim, and the process lasted up to 60 min. Interview transcripts that were in isiXhosa or Afrikaans were first transcribed before being translated into English using standard forward-back translation techniques. Participants were provided with a grocery voucher valued at ZAR100 (~US $8) for their time. 

### 2.1. Analyses

NVivo 11 was used to facilitate data analysis using the framework approach [33,34]. This analysis enabled a flexible process of capturing data under specific themes required for the research question, while allowing for new themes to emerge. This was particularly important in relation to prior life experiences and challenges that emerged from participants’ accounts of their counselling journey. The first (PPW) and second (CBS) author conducted the initial process of familiarization with the data through a review of transcripts. These authors discussed the initial framework and individually coded the first five transcripts. Following this, they discussed coding, major overarching themes and sub-themes and adjusted the framework. Coding then continued independently. Although all the transcripts were coded, no new codes emerged after 18 interviews were coded, suggesting thematic saturation. Any coding disagreements were resolved through discussion and consultation with the last author (BM). 

### 2.2. Ethics Approval

The South African Medical Research Council (EC 004–2/2015), the University of Cape Town (089/2015), and Oxford University (OxTREC 2–17) provided ethical approval for this study. The Western Cape Department of Health also approved this study (WC2016_RP6_9). The main trial is registered with the Pan African Clinical Trials Registry (Trial registration number: PACTR201610001825403). All participants provided consent to participate in the study.

## 3. Results

Our analyses revealed three major themes. The first related to participants’ inability to cope with adverse life experiences and stress and how maladaptive coping impacts drinking behavior and mental health; the second related to the perceived benefits of receiving a LHW-delivered MI-PST counselling program; and the third related to young women’s perceptions of various aspects of the MIND counselling program (see Table 2).

### 3.1. Sample Characteristics

The 27 women interviewed had a mean age of 28 years; 59.3% were Black African and 40.7% were colored (of mixed-race ancestry). Most women were single (59.3%) and most had only completed Grade 10 (29.6%) or Grade 11 (29.6%), with only two having completed high school. Most women were unemployed (85.2%) (see Table 3). At baseline, 26 of the 27 women met eligibility criteria for hazardous alcohol use. These women reported very harmful patterns of alcohol use (AUDIT: M = 27; SD = 10.3). All of the 27 women interviewed met eligibility criteria for depression—they reported a severe risk of depression (CES-D: M = 52.6; SD = 10.2). Almost three quarters of the women interviewed completed the core counselling program (70.4%).

### 3.2. Theme 1: Limited Ability to Cope with Adverse Life Experiences and High Levels of Stress Underpin Heavy Drinking and Depression

Participants frequently drew attention to adverse life experiences and current life stressors and described how their inability to cope with these experiences underpinned their heavy drinking and contributed to feelings of depression. More specifically, young women described a variety of complex and traumatic life experiences that they believed contributed to their depression and alcohol use. These included experiences of intimate partner violence (IPV) and sexual assault as well as HIV-related factors such as difficulty in accepting their HIV diagnosis, self-stigma and loss of self-esteem, experiences of stigma and discrimination from people in their community, loss of life partners/husbands and other family members (in some cases due to HIV).

*I lost the father of my child; after a week after his funeral, my child got shot but didn’t die…So, stress used to be a lot. [Participant 16]*

*The way I felt then [after diagnosis], it was as if I was going to commit suicide because my life was over and it couldn’t end quickly enough, so I’ll just put an end to it. [Participant 27]*

Young women also reported high levels of stress related to their current living environment, which they reflected contributed to their use of alcohol and feelings of depression. Several young women cited unemployment as their main source of stress, with others reporting concern about their children and substance abuse, guilt over the way they had treated their children, and worries about safety and substance use in their community.

*That was the biggest problem for me in my life. There were nights I couldn’t sleep, I’d worry if my child is dying…in and out of hospitals. Then I thought, your father is already dead now it’s you also. That’s the sort of things the devil was putting into my mind. If you die, you die. It’s because of him that I am sick, that sort of thing and it’s not fair that the child needs to suffer as a result of the sins of a parent. [Participant 1]*

Many participants reported using alcohol as a way of coping with these adverse life experiences and their stressful environment. While noting that drinking provided temporary relief from their problems, they acknowledged that heavy alcohol use had many detrimental effects. One participant attributed her ongoing exposure to IPV to both her own and her husband’s use of alcohol. Others reported that their children had been removed from their care by social services due to their harmful drinking, and another described an episode of extreme binge drinking when eight months pregnant.

For many of these participants, feelings of depression co-occurred alongside their heavy drinking and impacted on their adherence to ART. While a minority of participants reported good adherence to their ART despite experiencing problems in their lives, most participants reflected how their drinking and feelings of depression contributed to difficulties in remembering to take their ART as prescribed.

*I don’t want to lie…when I had depression and was drinking alcohol…I even forgot the pills and didn’t take them. I drank even if I knew that I had to take them, and I would be… “no, I will take them tomorrow”. [Participant 16]*

### 3.3. Theme Two: Perceived Benefits of the MIND Counselling

Participants described positive changes that arose from the counselling they received. They described improvements in HIV and mental health literacy; changes in the way they thought about themselves and their life experiences; positive behavior changes in their health and social lives; and a desire to support others with similar life experiences.

Most young women reported gaining a better understanding of their physical and mental conditions and the related importance of adherence to chronic medication through exposure to this counselling program. Several participants described their new understanding of how alcohol use, stress and depression impacted on the health of people living with HIV. Young women with depression, which is often undiagnosed, reported that the intervention enabled them to name and understand their experience.

*It was questions that were alright because they…how can I put it? They helped me ... understand that since I didn’t realise that I might have depression… at the same time, it opened my mind that oh no man… on how depression comes in order to have it…Because you are not aware that I have depression but when you are being told something, it becomes clear. [Participant 6]*

As a result of this understanding, most participants described adhering better to ART and reducing behaviors that may impact on their health such as smoking and alcohol use. Several participants described how using the problem-solving approach had enabled them to find practical ways to reduce their drinking; for example, drinking only on the weekends or drinking only at home where a limited amount of alcohol was available.

*I am taking my pills in the right way… I take them correctly now and no longer have that thing of drinking and skipping I…I have even gained weight. [Participant 4]*

*Sometimes I think about drinking again. Then I take my [intervention] booklet and page through it and read. That craving for wine is not there anymore. I am pregnant with my second baby and I told myself I do not want to drink anymore. [Participant 14]*

Participants described how the counselling had helped them replace drinking with other activities that reduced their isolation and represented steps towards improved social and occupational functioning. These activities included church going, joining women’s groups in their community, improving their education, taking up small-scale income-generating activities, and spending time on domestic responsibilities such as child care that they had previously neglected.

*It helped me with many things; like my children, my family and I started looking forward to my job. I was at work every day, started cutting off (drinking) friends. I was at home more often with my children. That’s something else I am proud of. [Participant 24]*

Young women also reported how the intervention content that focused on managing negative thoughts and accepting problems that they could not change had helped them reframe how they thought about their life circumstances. They felt that their newly acquired skills for limiting negative and intrusive thoughts helped them identify and limit catastrophic thinking and rumination about their circumstances which contributed to a sense of helplessness and despair. In addition, they felt that these aspects of the counselling program had helped them to think differently about their life circumstances, have greater acceptance of their HIV status, improve their self-esteem and sense of personal agency and resist the impact of stigma from others in their community.

*It taught me not to think that when I am in trouble everything is finished; there is nothing, no carrying on with life. It made me to be able to deal the problems I have. Even with stress. [Participant 10*]

*I used to be an angry person. Just because of how I got it, HIV. It [Project MIND] helped me a lot with quick anger, begrudging people thinking they judge you and blaming people for your reasons…. I can control it now. [Participant 9]*

A subgroup of young women described how building their own capacity for problem-solving enabled them to be a support and provide information to others around them, including parents, partners, extended family members and friends. One participant was able to assist her parents in getting help for their gambling addiction through a referral from the Project MIND counsellor. Others described being able to share information on HIV with her peers, particularly as it related to the impact of heavy drinking on adherence. One suggested the addition of a support group of peers with similar histories and health problems to the MIND intervention.

*I always give my friends advice from the booklet… then I say read the booklet then you will tell me again. And if she has read the booklet, then she comes back and says, you know, what is in the book is true. Then I say, yes girlfriend….I give people advice about this illness I have, she has the same illness….I tell her, drink your tablets then you will live for a long time. [Participant 13]*

### 3.4. Theme Three: Perceptions of LHW-Delivered Counselling

Most young women reported being satisfied with having an LHW provide counselling. They described how their counsellor provided them with support and comfort, highlighting the value of having a person listen to them with compassion and without judgement. One participant emphasized these benefits in helping her cope with her husband’s death.

*I felt comfortable, because what I noticed is that she does not judge…Instead of judging, she encourages…That made me to be free, to talk to anyone and not hide things [Participant 9]*

All the young women expressed positive views of their counsellors. They felt understood and reassured by counsellors enabling them to disclose stigmatized behaviors and adverse experiences. Young women commonly described the interaction with counsellors as being comfortable, understanding, and involving the exchange of humor.

*I won’t say she changed my life, but she made me a better person. I could talk to her about anything and because of her I am the person I am today. If it was not for her I would still be on the street, not worried about my child, still drinking. [Participant 14]*

The young women identified counsellor characteristics that enabled this positive interaction including being kind, empathetic, caring, friendly, supportive, easy to relate to, motivated and patient in helping them understand new concepts. One participant noted the importance of language/dialect for the quality of the counsellor-patient relationship.

Although a minority of participants experienced some initial anxiety and felt “nervous” and “fearful” at the beginning of the first counselling session about being asked personal questions, through the skills of the counsellor, all participants were able to open up and engage with the content of the program. Participants were given a booklet as part of the counselling sessions that summarized the content of each counselling session; most participants referred to these reading materials provided as helpful in supporting behavior change. They reported not only using this as a personal resource but also sharing it with others (e.g., partners).

*The thing that helped me the most is the book…Because once something happens, I would remember that it was said that, “When something like this occurs, I must open a particular place and read.” [Participant 7]*

Some did, however, report that they struggled to use the booklet as a resource between counselling sessions, either due to competing priorities or due to literacy issues. Although the program makes provision for participants who have literacy constraints, this remained a source of anxiety for some participants, as described by a participant who described feeling ashamed to inform her counsellor about her lack of literacy.

*I was thinking…should I talk or not…I was a bit fearful but it served me well afterwards. I could learn a lot. It helped me with many things; like my children, my family and I started looking forward to my job. [Participant 24]*

## 4. Discussion

This study describes young South African women on ART’s experiences and perceptions of an LHW-delivered MI-PST counselling program to reduce heavy drinking and symptoms of depression. The findings are among the first to highlight the mental health counselling needs of this vulnerable population at high risk for disengagement from care. 

More specifically, findings highlight the impact of traumatic experiences and stressful life circumstances on young women’s use of alcohol and risk of depression. Almost all young women described experiencing major traumatic life events. Underdeveloped coping skills and lack of social support seem to contribute to women becoming depressed and drinking as a way of numbing these feelings and coping with the stresses and strains of their unpredictable environment. This finding is in keeping with earlier South African studies that have shown high rates of substance use coping among South African women with histories of psychological trauma related to experiences of physical and sexual abuse [35]. It adds to what is already known by highlighting how receipt of an HIV diagnosis is often traumatic and how young women’s ability to accept and cope with this life-changing diagnosis is often constrained by adverse social circumstances and experiences of stigma [25,36]. This extends what we know about the need to strengthen the coping strategies of PLWH who drink to improve adherence to ART [25,37] by focusing on the role that trauma and environmental factors play in women’s drinking and feelings of depression. While previous studies have described how avoidant approaches to dealing with life stressors can lead to drinking and depression among PLWH [32,38], far less attention has been given to the role of traumatic life experiences and social circumstances. Although there have been some efforts to develop interventions that address traumatic stress among South African women who are living with HIV [39,40], these interventions have not included a focus on alcohol use and depression which intersect with experiences of trauma; other trauma-focused substance use interventions have been developed but have not focused on young women using ART specifically [35,41]. Our findings suggest that helping young women living with HIV to find ways of accepting problems and life circumstances that cannot change, including strategies for helping women manage their emotional responses to these experiences, is key to enabling them to move on with their lives, change their health risk behaviors, and optimize their use of ART. While young women in this study thought that the intervention was helpful even though it did not focus on traumatic stress, our findings of the salience of these traumatic experiences suggest the potential value of adapting the MI-PST intervention to include components that focus on traumatic stress and trauma responses.

Findings from this study also highlight the need to help women build personal agency to empower women to take charge of their health and move on from negative past experiences. The Joint United Nations Program on HIV/AIDS (UNAIDS) lists low personal agency (where women are unable to make choices and take action on matters of their own health and well-being) as one of the seven core reasons why adolescent girls and young women are more vulnerable to HIV than their male counterparts [42]. The focus on building personal agency in interventions aimed at this population is particularly important for women whose sense of agency has been diminished due to trauma or violence at the hands of others. Through this intervention, young women in this study felt empowered with new problem-solving skills to tackle aspects of their lives that were causing them stress. This impacted positively on their self-esteem and sense of agency. Components of the intervention that helped them manage negative and intrusive thoughts and accept problems or stressors that would never change (such as an HIV diagnosis) were experienced as especially empowering and gave women a sense of control over their lives. This led to changes in their thought patterns and health risk behaviors, including adherence to ART. The empowering effects of the intervention are further demonstrated through young women’s spontaneous efforts to share the intervention content with their peers and partners. Related to this, young women felt empowered enough to act as peer leaders, describing how they have been enabled to support others with similar life circumstances. The addition of peer support groups to the intervention was one of the recommendations that emerged from these participants who felt that these groups could provide a supportive context for young women as they tried to make behavioural changes within a challenging environment. The value of peer support groups in the treatment of substance use disorders [43], depression [44] and among PLWH [45] has been demonstrated. Similar to other studies [36], our findings highlight the role of peers and the importance of incorporating a peer support aspect in psychosocial interventions aimed at this population.

Finally, this study highlights the importance of the relationship between young women and the counselling delivery agent. In describing their relationships with counsellors, young women described individuals who were supportive, empathetic, non-judgemental, comforting, trustworthy and reassuring. Lay counsellors were chosen as intervention delivery agents given the emphasis on task-sharing mental health counselling to non-specialist providers in South Africa and similar low-and middle-income countries [23], patient preferences for lay counsellors [38], the feasibility of training lay counsellors to deliver this particular intervention [32] and evidence of the effectiveness of lay counsellor-delivered psychological interventions [46,47]. Our findings emphasize the value placed by young women on the relationship with the LHW and the importance of identifying lay counsellors who possess key qualities to effect change in a manner acceptable to young women during the hiring and training process. 

The findings from this study should be considered in the light of limitations synonymous with qualitative research. First, although this sample is largely representative of the young women who participated in the trial, the extent to which it is representative of the total population of adolescent girls and young women receiving ART for HIV in South Africa is not known. Second, we are unable to draw conclusions about the impact of this intervention on alcohol use, depression or adherence to ART. Third, while we took precautions to limit social desirability bias, participants may not have felt comfortable criticising the intervention. Finally, as a substantial number of participants selected for an in-depth interview were not contactable, our sample may have been skewed towards participants who felt the intervention was acceptable and beneficial. 

## 5. Conclusions

Despite some limitations, this study yielded valuable information to strengthen our understanding of the psychological intervention needs of young South African women on ART who report heavy drinking and/or depression. First, while young women seemed to find the intervention beneficial, our findings suggest that complex adverse life experiences are key drivers of young women’s use of alcohol and depression and impact on ART adherence. Future applications of this MI-PST intervention for this population may wish to consider adapting it to be trauma-focused, as this may improve its relevance for the lives of this vulnerable population. Second, it provides important insights into aspects of the MI-PST intervention that young women found most beneficial. The findings confirm that teaching women how to cope with these life circumstances, manage current stressors and accept past experiences is a valuable component of interventions for this population. Third, the findings highlight the importance of developing personal agency among young women and of including agency-building activities within counselling program. It also highlights the important role of peers in providing social support and in building agency for behavior change. Finally, the findings confirm the acceptability of LHW-delivered counselling for this population, provided that LHWs embody the personal characteristics that young women on ART value and respect. 

## Figures and Tables

**Table 1:** Summary of blended motivational interviewing–problem-solving therapy (MI-PST) sessions.

Session 1	Provide feedback on mental health assessment Increase knowledge of depression and alcohol use and their impact on the course of HIV (or diabetes) Identify a behavior to modify and use MI to build rapport and develop readiness to change Develop a change plan Describe Take Home Activity #1
Session 2 (50–60 min)	Patient check-in using MI Review activities from session 1 Build the rationale for PST Teach the steps of PST Conduct two problem-busting sessions Describe Take Home Activity #2
Session 3	Patient check-in using MI Review activities from session 2 *Coping with negative thoughts*: Explain how to cope with problems that are not important *Advance process of acceptance*: teach how to deal with problems that are important and cannot be solved Conduct a problem busting session
Booster Session	Patient check-in using MI Review of previous activities Conduct a problem busting session

**Table 2:** Process evaluation themes for the lay health-worker (LHW)-delivered MI-PST counselling program.

Major theme: Limited ability to cope with adverse life experiences and high levels of stress underpin heavy drinking and depression	Major Theme: Perceived benefits of the MIND counselling	Major Theme: Perceptions of LHW-delivered MI-PST counselling
Subtheme 1: Adverse life events: Complex trauma history *I used to never have peace with my past, like the thing that happened in my life…I told myself that I will never have a partner since I was abused a lot.*	Subtheme 1: Improved Knowledge and Health-Promoting Behavior Change *Yes, my medication, the tablets, he told me if I take my tablets it will be better for me and it will make a healthy baby. He advised me again it will be a healthy baby…then I will have the illness but she will not have it.*	Subtheme 1: Quality of relationship with counsellor *I discovered that it is useful in the sense that you can talk to someone who is not going to judge you…It is unlike when I speak to my mother and my sister, you see, my partner... It’s quite different because I am talking to someone else…And then she doesn’t do what?* *She doesn’t judge me.*
Subtheme 2: Current stressful life events and stressful environment *There were nights I couldn’t sleep, I’d worry if my child is dying…in and out of hospitals. Then I thought, your father is already dead now it’s you also.*	Subtheme 2: Improvements in thought patterns and attitudes to life circumstances *I thought like in life you should not stress…about the things that will take you nowhere that will gain you nothing. That is something I learnt and knew that it is important for my stress if I have stress, not to stress for too long…If there is, it must be present and then let it pass…What I liked the most in it is the fact that to me, it returned life firstly. Like I knew the fact that the place that I am in I must be satisfied. It taught me to be satisfied in the situation that I am in.*	Subtheme 2: Appropriateness of content and/or delivery *The book helped me a lot. I sometimes still use it when I feel depressed. Then I take my book and I page through it because I already filled in my answer in the book….no man, the book will still help me a lot in future when I feel down or alone.*
	Subtheme 3: Desire to Support Others *There are many people who have the virus, even my friends. They are still so young, then I tell them to stop drinking then they’ll see how they move forward and prosper.*	

**Table 3:** Socio-demographic characteristics of participants (n = 27).

	Frequency	Percent
**Race**	27	100
African	16	59.3
Colored (Mixed race)	11	40.7
**Age**	27	100
20 or less	2	7.4
21–29	12	44.4
30–35	13	48.2
**Marital Status**	27	100
Single (never married, divorced, widow)	19	70.4
Married/living with partner	8	29.6
**Highest Level of Education**	**27**	100
Diploma/other post school studies complete	1	3.7
Standard 10/Grade 12	1	3.7
Standard 8/Grade 10	8	29.6
Standard 9/Grade 11	8	29.6
Primary school	3	11.1
Standard 6/ Grade 8- Standard 7/ Grade 9	6	22.2
**Employment**	27	100
Employed full-time	3	11.1
Employed part-time	1	3.7
Unemployed	23	85.2
**Mental Health**	**MEAN**	**SD**
AUDIT	27	10.3
CES-D	52.6	10.2

## Appendix A. Interview Schedule for Patient Feedback

**Participant Satisfaction Form—End of Study Interview**

**Project MIND**

PARTICIPANT ID:………………………………………………………|__|__|__|__| SITE ID:……………………………………………………………………|__|__|__|__| STAFF ID:…………………………………………………………………………|__|__| DATE:………………………………………………|__|__| / |__|__| / |__|__|__|__| DD MM YYYY

*READ: Thank you for agreeing to talk with me about your experience with the Project MIND programme. Now that you have completed the counselling sessions, we want to ask you some questions about your experiences about the service you received. The information you provide us with will help us improve the intervention.*

**Study Experience                                                   **

A. What did you think about the questions that the counsellor asked you before starting the counselling (that is the screening questions about depression and alcohol use)?

*Probe*

1. What about the number of questions?
2. Were there any questions that were difficult to answer?

B. From what the counsellor explained to you, how much do you think your _________________ (*interviewer to fill in from what is known about participant: depression and/or alcohol use*) is making it difficult for you to follow the treatment programme for your _________________(*interviewer to complete: diabetes/HIV*)?

C. What would you like to tell us about your experience of the counselling?

*Probe*

1. What did you like most about the counselling programme?
2. What did you not like about the programme?
3. Were their parts that were difficult to understand or did not work well?

D. What did you learn from the counselling programme?

*Probe:*

1. Were there any new skills that you learned?
2. What new information did you learn?

E. Since you completed the counselling programme, what (if any) changes have you been able to make in your life?

*Probe*

1. Can you describe anything that you are doing differently in your life?
2. How has the counselling made a difference in your life?

F. How have you used what you learned about problem solving during counselling to your life? Can you give some examples of how/when you have put these skills to use?

*Probe:*

1. Please describe any specific problems that you have solved using these skills?
2. How have you continued to use the skills you learned in your daily life?

G. Were there any parts of the counselling programme that were less helpful to your life?

*Probe:*

1. __________________________________________In which ways could the intervention have been more helpful to you for your mental health and/or chronic disease.

H. How do you feel about the counsellor who provided the programme?

*Probe:*

1. How much did you feel the counsellor understood you?
2. Do you think the counsellor cared about your situation?
3. Was there anything that frustrated or upset you about the counsellor?

I.__________________________________________How comfortable do you feel during the sessions? What, if anything could we change to make you feel more comfortable?

K.__________________________________________What did you think about the number of sessions? Would you have liked more or fewer sessions with your counsellor?

M.__________________________________________What did you think of the patient handouts? What did you like the most? Is there anything you would have like to see changed?

*N*.__________________________________________How easy was it to attend the counselling sessions?

*Probe:*

1. Was there anything that made it difficult for you to keep your appointments with the counsellor?
2. Is there anything we can do to help people keep their appointments?

M. Do you have anything else to tell me about your experience of the counselling programme?

*Probe:*

1. What, if anything would have made your experiences of this programme better?

**Ending Questions                                                   **

Our time is about up. You have provided us with a lot of information in this short amount of time. Thanks again for your time—we really appreciate all of your help.

***[Give a short oral summary of the key ideas that emerged from the discussion.]***

A. Is this an adequate summary of the things that we have discussed today?

B. Do you have any questions for us?

C. Do you have anything to add that we may have missed?

_______________________________________________________________________________________________________________________
